# Effect of Bariatric Surgery on Diagnosed Chronic Kidney Disease and Cardiovascular Events in Patients with Insulin-treated Type 2 Diabetes: a Retrospective Cohort Study from a Large UK Primary Care Database

**DOI:** 10.1007/s11695-019-04201-y

**Published:** 2020-03-04

**Authors:** Mohammed Alkharaiji, Uchenna Anyanwagu, Richard Donnelly, Iskandar Idris

**Affiliations:** 1 Division of Medical Sciences & Graduate Entry Medicine, School of Medicine, Royal Derby Hospital, University of Nottingham, Derby, UK; 2 Faculty of Public Health, College of Health, The Saudi Electronic University, Riyadh, Saudi Arabia

**Keywords:** Bariatric, Diabetes, Kidney, Renal, CKD, Microalbuminuria, Obesity, Cardiovascular

## Abstract

**Aims:**

To compare the effect of bariatric surgery on renal, chronic kidney disease (CKD) and cardiovascular (CV) outcomes among obese patients with insulin-treated type 2 diabetes (T2D) with and without microalbuminuria (i.e., uACR > 3.0 mg/mmol).

**Methods:**

A retrospective cohort study was conducted among 11,125 active patients with T2D from The Health Improvement Network (THIN) database. Propensity score matching (up to 1:6 ratio) was used to identify patients who underwent bariatric surgery (*N* = 131) with a non-bariatric cohort (*N* = 579). Follow-up was undertaken for 10 years (6487 person-years) to compare differences in risk of cardiovascular events and in renal outcomes.

**Results:**

For the matched cohort at baseline: mean age 52 ± 13 years (60% female); weight 116 ± 25 kg, body mass index (BMI) 41 ± 9kg/m^2^, estimated glomerular filtration rate (eGFR); 70.4 ± 20 mL/min/1.73 m^2^, and median albumin-creatinine ratio (uACR) 2.0 mg/mmol (interquartile range (IQR): 0.9–5.2 mg/mmol). Bariatric surgery was associated with a 54% reduction in developing CKD compared to their matched non-bariatric cohort (adjusted hazard ratio [aHR]: 0.46; 95%CI: 0.24–0.85, *P* = 0.02). Among patients with microalbuminuria at baseline, bariatric surgery was protective against CKD (aHR: 0.42, 95%CI: 0.18–0.99, *P* = 0.050). eGFR was significantly increased from baseline favouring the bariatric group during 75% of the follow-up time (calculated mean difference between groups: 4.1 mL/min/1.73 m^2^; *P* < 0.05), especially at 5-year point (74.2 vs 67.8 mL/min/1.73 m^2^; *P* < 0.001). However, no significant change was observed with non-fatal CVD episodes (aHR: 0.36, 95%CI: 0.11–1.13, *P* = 0.079). Albumin levels were significantly reduced throughout the 2 years following the surgery (3.9 vs 4.1 g/dL, *P* < 0.001). uACR and total protein levels had little or no statistical association to the intervention.

**Conclusion:**

Bariatric surgery may protect patients with diabetes with or without microalbuminuria against the risk of CKD and with a modest protective effect on non-fatal CVD risk. Bariatric surgery is also associated with improvements in overall renal outcomes such as eGFR.

## Background

Obesity and type 2 diabetes (T2D) are major global health problems that are intrinsically linked with adverse cardio-renal outcomes [1, 2]. Dysfunctional adipose tissue in obesity is associated with increased pro-inflammatory state, insulin resistance, hyperglycaemia, endothelial dysfunction and hypertension, all of which are known risk factors for the development and progression of cardiovascular (CV) disease and chronic kidney disease (CKD) [2, 3]. Furthermore, many patients with T2D will require insulin treatment to control hyperglycemia. This is relevant within the context of diabetic kidney disease since insulin therapy is known to induce ~ 4–9 kg weight gain in the first year of treatment [4] while obesity per se is a significant risk factor for the appearance of proteinuria and ESRD [2]. Further, recent evidence from randomised controlled trials, epidemiological and observational studies have implicated insulin therapy in patients with T2D with increased CV risk and mortality [5–8]. Thus, a cohort of insulin-treated patients with T2D represents a complex heterogenous, challenging group of patients, many of whom have significant comorbidities and high CKD risk. CKD is defined as an estimated glomerular filtration rate (GFR) of less than 60 mL/min/1.73 m^2^ or the presence of increased urinary albumin excretion (microalbuminuria), indicated by urine albumin-to-creatinine ratio [uACR] of 3.0–30.0 mg/mmol), or overt proteinuria (uACR > 30.0 mg/mmol), all of which are independent risk factors for CV and kidney disorders in the general population and in patients with diabetes [9]. Thus, weight loss by any means is important to improve cardio-renal outcomes [10]. Although diet and exercise play a crucial role in obesity management, lifestyle alone may not achieve durable weight loss in the majority of patients [11]. Bariatric surgery, therefore, has emerged as the most effective and durable strategy for long-term weight loss in morbidly obese individuals [12].

Despite the clear benefits of bariatric surgery on weight and glycemic outcomes in people with T2D, the impact of bariatric surgery on the development and progression of CKD or micro-albuminuria is less clear. Previous studies have reported improvements in uACR [13–16], which can be observed not long after surgery [15, 16]. This is thought to be driven by multi-factorial improvements in blood pressure, HbA1c and BMI [15]. A further study concluded that bariatric surgery should be offered as an early treatment for patients with microalbuminuria or with overt proteinuria to prevent CKD from progressing to an end-stage kidney disease (ESKD) [17]. However, many of these studies were either small case series, not specific to people with insulin-treated diabetes, had variable albuminuric state at baseline, or did not adjust for important confounders. Conversely, inconsistent findings were reported in a systematic review of the effects of bariatric surgery on renal outcomes by Zhou et al [18], with some studies also noting harmful effects on kidneys of obese patients who received bariatric intervention. For example, there may be an increased risk of kidney stones after malabsorptive bariatric surgery, which is considered to be linked with surgery-induced fat malabsorption [19]. Furthermore, despite weight benefits, there are negative metabolic outcomes related to bariatric intervention, such as nutritional deficiencies, reduction in lean body mass and bone density loss, all of which are highly relevant in patients with CKD [20].

This study aims to retrospectively explore the possible preventive effect of bariatric surgery against CKD, renal-cardiovascular, as well as impact on health and renal outcomes in patients with and without microalbuminuria (i.e., uACR > 3.0 mg/mmol).

## Methods

### Study Design and Data Sources

This was a retrospective cohort study that used The Health Improvement Network (THIN), a UK primary care database with systematically computerised longitudinal and anonymised health records from primary care physicians. The database contains details on demographics, lifestyle characteristics (e.g. alcohol use and smoking), major medical and surgical conditions, drug utilisation and various health outcomes of over 17 million patients, 3.1 million of which are registered as active patients. Patients’ records were derived from over 600 UK general practices [21]. The dataset slice we acquired has information of active patients with insulin-treated type 2 diabetes up to September 2017. Ethics approval was provided to THIN by the NHS South East Multi-Centre Research Ethics Committee (MREC). The Scientific Review Committee (SRC) reviewed the study protocol for scientific merit and feasibility.

### Study Population

The dataset contains 11,125 adult patients (18 years and over with no upper age limit) diagnosed with T2D who had been prescribed with any form of insulin therapy up to September 2017. Patients’ index date was based on either the day of bariatric surgery or, in case they had not received bariatric intervention, the first intensification of insulin therapy. Dataset was scanned to identify patients with no history of insulin use or diagnosed with type 1 diabetes for possible exclusion.

### Exposure and Outcomes

Our exposure of interest is bariatric surgical intervention for morbidly obese patients with insulin-treated T2D. The study was exposure-based, in which patients were censored throughout 10 years of follow-up—following the development of primary outcome, transferred out, loss to follow-up, or at the end of the study. Primary outcomes were patients’ survivability against diagnosed CKD events, with further stratification to include CKD and CVD events, from a population of patients with microalbuminuria (uACR > 3 mg/mmol) at baseline. The risk of CKD was measured according to observed CKD data that were reported with specific dates in the main THIN database. The CKD events were assessed and reported by health professionals according to the medical criteria used across the NHS primary health provision (GFR < 60 or ACR > 3.5) and covered by the THIN database collection system. The crude CVD events included the first occurrence of either acute myocardial infarction, stroke, coronary heart disease, heart failure, or peripheral artery disease. Observations of CVD events were obtained in a similar manner as with events of CKD reporting system prearranged by THIN. Secondary outcomes included likelihood of being improved in eGFR, as well as health covariates, such as levels of uACR, total protein, albumin and serum creatinine.

### Covariates and Follow-up Strategy

The treatment group, those undergoing bariatric surgery and being insulin-treated T2D from the date of surgery, was followed up and compared with their propensity-score (PS)-matched insulin initiators from their first insulin prescription date up to the endpoint of the 10-year follow-up period. Patients with diagnosed CKD or whose CVD events occurred prior to the designated baseline point were excluded from the primary survival estimation on each stratified element.

The baseline clinical parameters were measured at a similar point of time according to patient’s treatment category. Patients who underwent bariatric surgery, for instance, will have their baseline parameters calculated^1^ from 90 days up to 1 day before the surgery date. Similarly, the non-bariatric patients will have their baseline parameters calculated via the same time window according to their first intensification of insulin therapy. Covariates were, then, recalculated at 6 months, and at each year point during follow-up, with a 90-day window on every concurring point of time.

### Statistical Analysis

Primary analysis was time to the risk of crude CKD events and stratified CKD and CVD events in the PS-matched groups. The PS model was estimated by using a logistic regression model in order to adjust for baseline characteristics, thus, minimising allocation bias between groups. The balance assessment was made between bariatric (treated) and non-bariatric (untreated) groups by measuring standardised differences before and after the matching procedure. The mean from continuous covariates and proportion of categorical variables between groups were examined and summarised. Each treatment subject was matched to a maximum number of six reference subjects at the nearest distance measured by the estimated PS, based on the estimated treatment probabilities [22]. Furthermore, in order to minimise distance within matched sets, we employed calliper of width equal to 0.05 of the standard deviation of the logit of the PS, which may improve match quality and also limited excessive numbers of matched subjects [23]. A calliper of width of 0.2 or lower was shown in recent research to result in optimal estimation compared to higher choices of calliper use [24]. PS was included in all Cox proportional hazards regression modelling as it was considered a prognostic covariate.

We used the stratified log-rank test, with Kaplan-Meier survival curves, respectively, to compare the equality between the PS-matched groups. The absolute reduction in the probability of an event occurring within 10-year follow-up was, thus, calculated. Marginal hazard ratios were also estimated, which allowed the quantification of the adjusted hazard of an event occurring in the bariatric group compared to the matched non-bariatric group. Proportional hazards assumptions were confirmed through Schoenfeld residual test. Point estimates with 95% confidence intervals (CIs) at the conventional statistical significance level of 0.05 were used in the regression models. The proportional hazards assumption was examined by comparing the cumulative hazard plots grouped on exposure; no violations were observed.

Missing data among covariates were managed through multiple imputations using the predictive means matching for continuous covariates with accounting to exposure (i.e. bariatric), age, gender, diabetes duration, Townsend deprivation status, marital status, smoking and alcohol use [25]. To test the adequacy of our multiple imputation approach in addressing the impact of some missing data, we conducted a sensitivity analysis wherein the primary endpoints in the imputed dataset were compared with the dataset with missing values and found to be similar up to 2 years of follow-up. This affirmed the robustness of the imputation method employed before PS matching procedure was performed [26].

We used Student’s *t* test to estimate the mean changes in continuous variables (e.g. uACR and total protein) in the PS-matched group for 2 years of follow-up compared to their baseline measurements. We limited these variables to 2 years due to the heavy load of missing data beyond this point, which restricted multiple imputation from producing reliable predictions. Nonetheless, eGFR was at a predictable level up to 5 years, which made it possible to run Pearson *X*^2^ to test the likelihood of being improved throughout 5 years from the baseline. Wilcoxon rank-sum test was used to check differences in medians for nonparametric variables (i.e. uACR). Statistical significance was set at a *p* level of 0.05. To avoid the probability of type II error, the study was powered to 0.86 and the matched sample size of 710 was found to detect a true difference of less than 0.1 between the two groups at 5% significance level. The study fulfilled the STROBE criteria for reporting observational studies. Throughout, we used SAS Software version 9.4 in the initial dataset management (SAS Institute, Cary, NC); Stata/SE Statistical Software version 15.1 in all carried analysis (StataCorp., College Station, TX); and GraphPad/Prism version 8.1.0 for visualisation (La Jolla, CA).

## Results

### Patients’ Characteristics and Total Follow-up

From a total population of 11,125 patients with insulin-treated T2D in THIN database, we identified 155 patients who have had Roux-Y-gastric bypass surgical operations. The PS matching procedure has allowed (131) bariatric patients to be matched with up to six control subjects (579). This yielded a total number of 710 PS-matched participants. The median treatment duration was 10.07 years (interquartile range (IQR): 6.11–14.31 years). The median follow-up was 12.8 years (IQR: 5.1–14.5 years) for the matched cohort, representing a total follow-up period of 6487 person-years.

The mean age for the matched groups at baseline was 51.7 (SD 12.5) years; 59.6% were females. The mean body weight, BMI and HbA1c level were 115.7 (SD 25.4) kg, 40.7 (SD 9.2) kg/m^2^ and 71.2 (SD 18.1) mmol/mol, respectively. The mean eGFR was 70.4 (SD 20.5) mL/min/1.73 m^2^, with a median uACR of 2.0 (IQR: 0.9–5.2) mg/mmol. The baseline characteristics in both bariatric and non-bariatric groups were compared between the full and matched cohort with respective standardised differences and are shown in Table 1.

**Table 1:** Baseline characteristics

	Cohort					
	Full population [*N* = 11,125]			Propensity matched [*N* = 710]		
Baseline variable	Bariatric [*n* = 155]	Non-bariatric [*n* = 10,970]	Std. diff^a^	Bariatric [*n* = 131]	Non-bariatric [*n* = 579]	Std. diff^b^
Demographics						
Age (years), mean (SD)	50.01 (11.1)	57.71 (13.3)	− 0.694	50.74 (11.0)	51.96 (12.8)	− 0.110
Gender, no (%)						
Female	89 (57.4)	5068 (46.2)	0.224	73 (55.4)	351 (60.6)	− 0.107
Townsend deprivation, %						
Least deprived	14.0	21.7	− 0.204	15.7	17.3	− 0.044
Less	24.3	20.7	0.086	24.0	18.1	0.145
Average	17.6	21.4	− 0.094	16.5	20.2	− 0.094
More	20.6	20.9	− 0.008	21.5	27.7	− 0.144
Most deprived	23.5	15.3	0.209	22.3	16.8	0.14
Type 2 diabetes (years), mean (SD)						
Diabetes duration	14.15 (7.7)	15.12 (8.4)	− 0.125	13.97 (7.8)	14.89 (7.6)	− 0.117
Insulin dependency	6.97 (4.9)	7.99 (5.5)	− 0.208	7.13 (4.9)	8.55 (5.6)	− 0.289
Drug use duration (years), mean (SD)						
Oral antidiabetics	11.61 (5.8)	10.77 (6.1)	0.145	11.89 (5.7)	11.26 (5.7)	0.110
Antihypertensive	12.69 (6.6)	12.21 (6.6)	0.074	12.89 (6.7)	12.25 (6.3)	0.095
Diuretics	8.22 (7.3)	8.99 (7.0)	− 0.105	8.09 (7.3)	9.32 (7.1)	− 0.168
Aspirin	8.34 (5.7)	8.69 (5.4)	− 0.061	8.64 (5.9)	8.25 (5.4)	0.066
Clinical parameters, mean (SD)						
Weight (kg)	127.3 (30.3)	90.79 (20.6)	1.204	123.22 (28.3)	114.88 (24.5)	0.294
Height (m)	1.7 (0.1)	1.68 (0.1)	0.201	1.7 (0.1)	1.69 (0.1)	0.102
BMI (kg/m^2^)	43.87 (10.0)	32.37 (7.5)	1.150	42.77 (9.6)	40.6 (9.0)	0.226
HbA1c (mmol/mol)	72.34 (19.3)	70.03 (17.2)	0.119	72.41 (18.6)	70.91 (17.9)	0.080
Fasting glucose (mmol/L)	9.83 (4.3)	9.93 (3.9)	− 0.023	9.84 (4.3)	9.82 (3.9)	0.004
Blood glucose (mmol/L)	12.22 (8.8)	11.69 (5.3)	0.071	12.04 (9.1)	11.92 (5.3)	0.016
SBP (mmHg)	134.64 (14.6)	138.89 (16.5)	− 0.271	135.06 (14.5)	136.4 (16.0)	− 0.088
DBP (mmHg)	78.66 (8.4)	78.94 (9.6)	− 0.031	79.3 (8.5)	78.77 (9.3)	0.058
Albumin (g/dL)	3.96 (0.4)	4.15 (0.5)	− 0.368	3.96 (0.4)	3.96 (0.4)	− 0.005
Alkaline phosphatase (IU/L)	98.31 (47.1)	91.62 (43.0)	0.146	98.79 (48.8)	96.88 (51.5)	0.038
Serum creatinine (μmol/L)	91.74 (78.4)	92.68 (52.6)	− 0.014	92.29 (84.0)	88.17 (57.7)	0.056
Triglyceride (mmol/L)	2.33 (1.5)	2.03 (1.3)	0.2	2.34 (1.6)	2.26 (1.4)	0.049
Total cholesterol (mmol/L)	4.47 (1.2)	4.49 (1.1)	− 0.019	4.52 (1.2)	4.52 (1.2)	0.002
Low density lipoprotein (mmol/L)	2.39 (0.9)	2.39 (0.9)	0.001	2.39 (0.9)	2.44 (1.0)	− 0.05
High density lipoprotein (mmol/L)	1.07 (0.3)	1.22 (0.4)	− 0.439	1.07 (0.3)	1.1 (0.3)	− 0.091
Alcohol status, %						
Unknown	3.7	3.1	0.03	3.3	3.0	0.017
Ex-drinker	11.8	7.0	0.162	11.6	11.5	0.003
Never	33.1	31.3	0.039	33.1	33.1	− 0.002
Current	51.5	58.5	− 0.143	52.1	52.4	− 0.006
Smoking status, %						
Ex-smoker	33.1	37.1	− 0.085	31.4	36.9	− 0.116
Never	52.9	49.7	0.064	52.9	52.2	0.015
Current	14.0	13.1	0.025	15.7	10.9	0.141
Comorbidities, %						
Hypoglycaemia	32.4	29.9	0.054	31.3	26.1	0.116
NAFLD^c^	4.7	3.0	0.090	4.6	2.8	0.097
Anaemia	15.5	12.2	0.096	16.8	13.3	0.098
Acute myocardial infarction	24.3	20.3	0.095	23.1	20.2	0.073
Stroke	11.0	12.9	− 0.059	12.4	7.7	0.156
Heart failure	18.4	17.8	0.016	17.4	18.5	− 0.029

Diabetes duration is time from first diagnosis of diabetes to date of intensification with insulin drug (index date)

^a^Standardised differences are the absolute difference in means or percentages divided by the SD of the treated group. Resulting standardised difference after 1:6 matching based on average treatment effect on treated propensity score technique and robust variance estimation

^b^Mean of standardized difference after matching (0.081), i.e. at 8% difference measured between the matched groups

^c^Non-alcoholic fatty liver disease

### Probability of Survival and Event Rate

The probability of survival for CKD in the full cohort was significantly different between bariatric and non-bariatric groups: at 1 year (99.2% vs 97.7%), 5 years (96.9% vs 89.9%) and 10 years (94.9% vs 80.2%) of follow-up (log-rank test *p* value = 0.028). However, the estimates of CKD event rate in the unadjusted PS matched cohort showed little or no statistical significance of a difference throughout 10 years of follow-up (log-rank test *p* value = 0.19). A total of 119 CKD events were observed (16 vs 103) with a crude event rate of 18.3 (14.5 vs 19.1) per 1000 person-years (95%CI: 15.3–21.9).

The difference in probability of survival for CKD in patients with microalbuminuria was statistically insignificant in both full and matched cohorts (log-rank test: *p* value = 0.14 and *p* value = 0.24, respectively). In the matched group, a total of 51 CKD events were observed (8 vs 43) with a crude event rate of 22.2 (14.3 vs 25.4) per 1000 person-years (95%CI: 17.2–29.8).

**Fig. 1:**
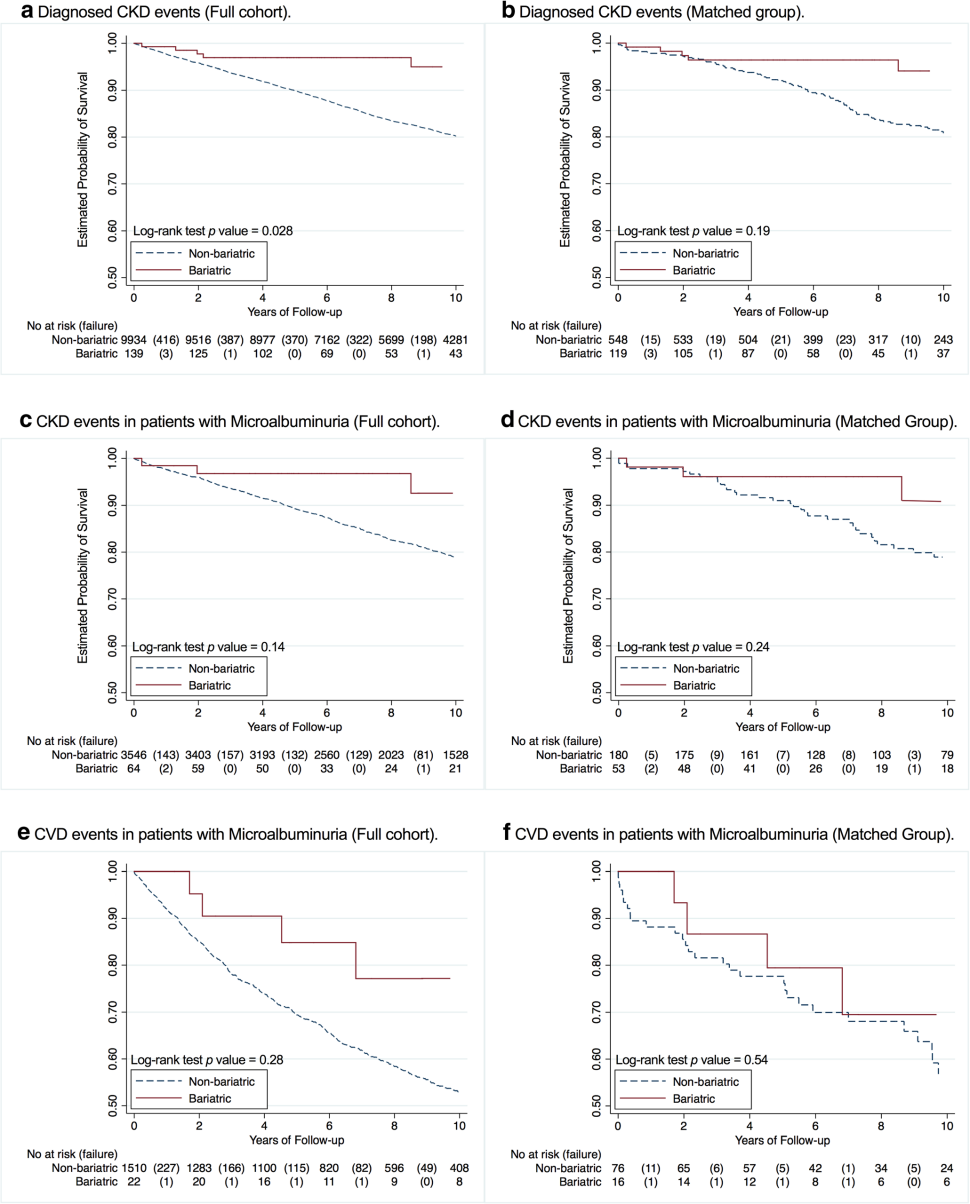
Bariatric vs. non-bariatric Kaplan-Meier survival analysis plots for diagnosed CKD events in **a** full and **b** matched cohorts, diagnosed CKD events in patients with microalbuminuria (i.e. uACR > 3 mg/mmol) at baseline in **c** full and **d** matched cohorts, and crude CVD events in patients with microalbuminuria at baseline in both **e** full and **f** matched cohorts throughout 10 years of follow-up

The difference in probability of survival for CVD in patients with microalbuminuria was statistically insignificant in both full and matched cohorts (log-rank test: *p* value = 0.28 and *p* value = 0.54, respectively). In the matched group, a total of 43 CVD events were observed (10 vs 33) with a crude event rate of 55.0 (49.5 vs 56.9) per 1000 person-years (95%CI: 40.8–74.2). Figure 1 and Table 2 summarise the observed events, event rates and differences in the probability of survival.

**Table 2:** Survivability of (A) patients against crude CKD events, (B) CKD events in patients with microalbuminuria at baseline as well as (C) CVD events and their respective crude incidence rates and hazard ratios of events in the full cohort and in the matched group

Survival analysis	Non-bariatric	Bariatric
A. Crude CKD events		
Full cohort, n	9934	139
Events/person-years, n	2032/96,843	16/1283
Absolute rates^a^ (95% CI)	20.9 (20.1–21.9)	12.5 (7.6–20.3)
HR^b^ (95% CI)	1 (reference)	0.56 (0.33–0.95)^†^
aHR^c^ (95% CI)	1 (reference)	0.53 (0.30–0.91)^†^
Matched cohort, n	548	119
Events/person-years, n	103/5385	16/1102
Absolute rates (95% CI)	19.1 (15.8–23.2)	14.5 (8.9–23.7)
HR (95% CI)	1 (reference)	0.67 (0.37–1.22)
aHR^d^ (95% CI)	1 (reference)	0.46 (0.24–0.85)^†^
B. CKD in patients with microalbuminuria		
Full cohort, n	3546	64
Events/person-years, n	775/34,517	8/650
Absolute rates (95% CI)	22.4 (20.9–24.1)	12.3 (6.1–24.6)
HR (95% CI)	1 (reference)	0.59 (0.29–1.20)
aHR (95% CI)	1 (reference)	0.71 (0.33–1.5)
Matched cohort, n	180	53
Events/person-years, n	43/1694	8/558
Absolute rates (95% CI)	25.4 (18.8–34.2)	14.3 (7.2–28.7)
HR (95% CI)	1 (reference)	0.62 (0.28–1.39)
aHR (95% CI)	1 (reference)	0.42 (0.18–0.99)^†^
C. CVD in patients with microalbuminuria		
Full cohort, n	1510	22
Events/person-years, n	710/11,304	10/258
Absolute rates (95% CI)	62.8 (58.3–67.6)	38.7 (20.8–72.0)
HR (95% CI)	1 (reference)	0.70 (0.37–1.34)
aHR (95% CI)	1 (reference)	0.30 (0.09–0.96)^†^
Matched cohort, n	76	16
Events/person-years, n	33/580	10/202
Absolute rates (95% CI)	56.9 (40.5–80.0)	49.6 (26.7–92.2)
HR (95% CI)	1 (reference)	0.77 (0.34–1.75)
aHR (95% CI)	1 (reference)	0.36 (0.11–1.13)

^a^Absolute rate at 1000 person-years

^b^HR (unadjusted hazard ratio)

^c^aHR (adjusted hazard ration). Adjusted for age, diabetes duration, duration of antihypertensive drug use, diuretics use, antidiabetic drug use (i.e. Premix) and deprivation (Townsend) status

^d^Adjusted for age, diabetes duration and insulin drug use

^†^*P* < 0.05 (probability reference)

### Risk of CKD

Bariatric surgery showed remarkable protective effect against crude CKD in the full cohort and in the adjusted matched group. In the full cohort, patients whom had been treated with bariatric surgery had 47% lower risk to develop CKD compared to non-bariatric patients (adjusted hazard ratio (aHR): 0.53, 95%CI: 0.30–0.91, *P* = 0.021). Similarly, the matched cohort showed a statistical significance of a magnitude favouring the bariatric group with a protective effect of 54% against crude CKD risk (aHR: 0.46, 95%CI: 0.24–0.85, *P* = 0.013). Table 2A shows a summary of adjusted and unadjusted hazard ratios in crude CKD risk for matched and unmatched patient groups.

### Risk of CKD in Patients with Microalbuminuria

Despite a protective tendency against CKD, patients with microalbuminuria at baseline have little or no statistical evidence of a similar protective effect in the full cohort (aHR: 0.71, 95%CI: 0.33–1.5, *P* = 0.38). However, the estimates imply a protective influence against CKD favouring the bariatric group in the matched cohort (aHR: 0.42, 95%CI: 0.18–0.99, *P* = 0.050). The adjustments made for this model have only omitted 13.2% of observed events. Table 2B shows a summary of adjusted and unadjusted hazard ratios in CKD risk for patients with microalbuminuria.

### Risk of CVD in Patients with Microalbuminuria

In the full cohort, patients with microalbuminuria, who had been treated with bariatric surgery, had a 70% lower risk in developing CVD (aHR: 0.30, 95%CI: 0.18–0.96, *P* = 0.043). The added adjustments for this model have omitted 37.1% out of the unadjusted model. However, these adjustments helped to reveal evidence of little or no statistical effect of such protection against CVD in the matched cohort (aHR: 0.36, 95%CI: 0.11–1.13, *P* = 0.079). Table 2C shows a summary of adjusted and unadjusted hazard ratios in CVD risk for patients with microalbuminuria.

### Change in Secondary Outcome Variables

Significant reductions in the matched cohort (i.e. *P* < 0.001) favouring the bariatric group vs non-bariatric were observed in terms of body weight and BMI throughout 5 years of follow-up time compared to baseline. Body weight and BMI for bariatric vs non-bariatric were at 1-year point (97.5 ± 24.2 vs 109.8 ± 18.6 kg; 34.2 ± 9.0 vs 38.8 ± 7.4 kg/m^2^, respectively), at 3-year point (95.7 ± 19.4 vs 108.8 ± 18.4 kg; 33.5 ± 7.4 vs 38.3 kg/m^2^, respectively) and at 5-year point (98.9 ± 23.3 vs 107.1 ± 18.2 kg; 34.8 ± 9.2 vs 37.8 ± 7.3 kg/m^2^, respectively).

The nonparametric tests for the uACR medians revealed little or no statistical significance of a difference between bariatric and non-bariatric groups in both matched and full cohorts. In the full cohort, the median uACR in bariatric group at baseline was 2.0 vs 1.91 mg/mmol in non-bariatric (*Z* = − 1.28, *P* = 0.19), at 1-year point 2.33 vs 1.90 mg/mmol (*Z* = − 1.86, *P* = 0.06), and at 2-year point 2.42 vs 2.06 mg/mmol (*Z* = − 0.87, *P* = 0.38), respectively. In the matched cohort, the median uACR in the bariatric group at baseline was 2.03 vs 1.90 mg/mmol in the non-bariatric group (*Z* = − 1.75, *P* = 0.08), at 1-year point 2.31 vs 1.95 mg/mmol (*Z* = − 1.36, *P* = 0.17) and at 2-year point 2.42 vs 2.02 mg/mmol (*Z* = − 0.67, *P* = 0.50), respectively.

**Fig. 2:**
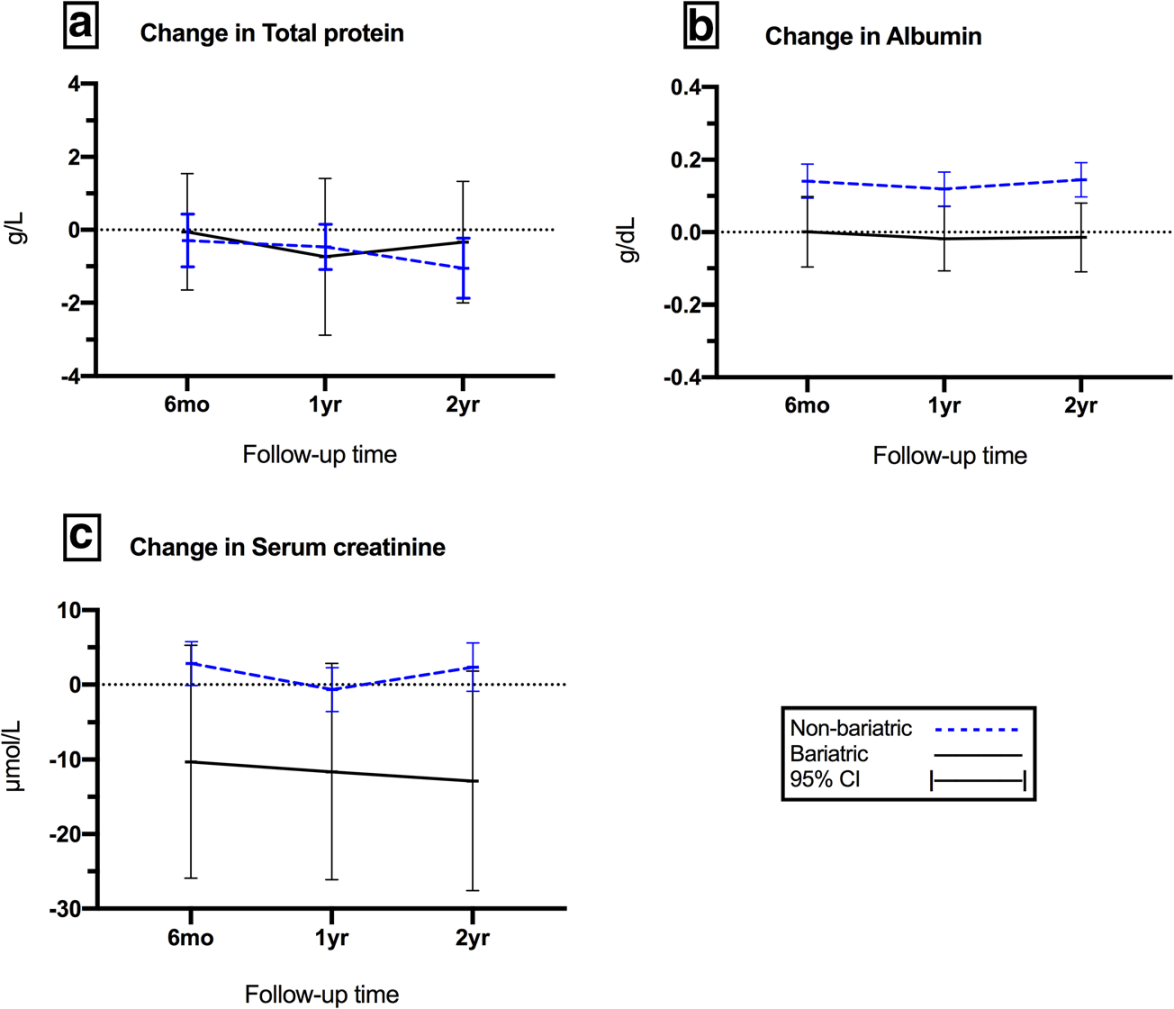
Mean change in **a** total protein (g/L), **b** albumin (g/dL), and **c** serum creatinine (μmol/L) in the matched groups, bariatric vs non-bariatric, compared to baseline

**Fig. 3:**
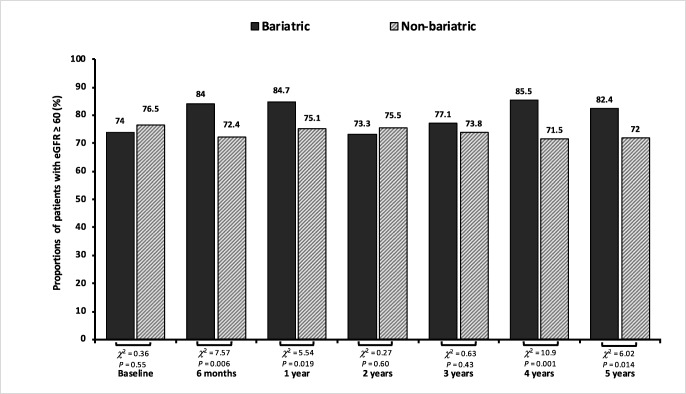
Proportions of patients (%) in the matched cohort with eGFR ≥ 60 (mL/min/1.73 m^2^) throughout 5 years of follow-up

There have been significant improvements in eGFR throughout 5 years of follow-up favouring the bariatric group in both full and matched cohorts. In the matched cohort, the eGFR was at similar levels at baseline with a mean of 68.7 in bariatric patients vs 70.8 mL/min/1.73 m^2^ in non-bariatric (*t*_708_ = 1.05, *P* = 0.29). Mean eGFR for the bariatric compared with non-bariatric group were 72.4 vs 68.4 mL/min/1.73 m^2^ (*t*_708_ = − 2.07, *P* = 0.038) at 1 year and 71.4 vs 68.4 mL/min/1.73 m^2^ (*t*_708_ = − 1.48, *P* = 0.13) at 3 years. However, during the fourth and fifth years of follow-up, the analysis of mean differences reported statistical significance favouring the bariatric group versus non-bariatric with 72.9 vs 66.8 mL/min/1.73 m^2^ at 4-year point (*t*_708_ = − 3.14, *P* = 0.001) and with 74.2 vs 67.8 mL/min/1.73 m^2^ at 5-year point, respectively. Figure 3 illustrates proportions of both bariatric and non-bariatric patients with eGFR more than 60 mL/min/1.73 m^2^ during 5 years of follow-up. The serum creatinine was also significantly reduced in the bariatric group compared to their matched non-bariatric counterparts during the 2 years of follow-up—following the baseline point. Both groups were at similar levels of serum creatinine at baseline with a mean of 90.1 (SD 84.1) μmol/L in bariatric versus 88.4 (SD 57.7) μmol/L non-bariatric (*t*_708_ = − 0.27, *P* = 0.78). Mean creatinine for the bariatric group vs non bariatric was 79.7 vs 91.2 μmol/L (*t*_708_ = 2.59, *P* = 0.009) at 6 months, 78.4 vs 86.1 μmol/L (*t*_708_ = 2.11, *P* = 0.03) at 1-year point, and 77.2 vs 90.5 μmol/L at 2-year point (*t*_708_ = 2.65, *P* = 0.008) (Fig. 2c).

In the matched cohort, the bariatric group had significantly lower albumin levels compared to non-bariatric throughout 2 years of follow-up. The total protein level showed a slight clinical change with a statistically significant difference at 1-year point, but with no difference detected in all other points of follow-up time. Figure 2 shows mean differences between the matched groups while reflecting back to baseline observations for (a) total protein and (b) blood albumin.

## Discussion

This study focused on severely obese patients with insulin-treated T2D and noted that there is a relationship between bariatric surgical intervention and protective effect against CKD and observed improvements in overall patients’ renal outcomes, with benefits reaching patients with or without microalbuminuria at baseline. Despite the matched cohort showing little or no statistical significance regarding protection against the risk of non-fatal CVD, the estimates following the surgery suggest a positive influence with lower event rate favouring the bariatric group. The survival analysis on full cohort also indicated a profound effect protecting microalbuminuria patients who had received bariatric intervention treatment concerning crude non-fatal CVD events. An overall improvement in the bariatric matched group regarding eGFR levels was also noted throughout 5 years of follow-up (Fig. 3).

We believe our findings are robust. A review of previously published studies has shown that we utilised a relatively novel approach that included an indirect assessment of time-to-event based on baseline patients’ renal status. This timely phase involved full, as well as the PS-matched cohorts, being allocated into further stratifications, leading to proper follow-up for survival investigation. In this approach, the bariatric surgical intervention provides additional evidence of a protective effect benefiting T2D patients with or without detected microalbuminuria at baseline.

Obesity is associated with glomerular hyperfiltration, and therefore increased risk of microalbuminuria and/or proteinuria in patients with or without renal disease [27, 28]. Previous studies have shown that bariatric surgery is associated with a reduction in glomerular hyperfiltration [29, 30] which mainly occurred in the first year after surgery being associated with the greatest period of weight loss. The link between fat mass and glomerular hyperfiltration is multifactorial, but partly due to increase of angiotensin II (AngII), which enhances tubular sodium reabsorption and activates tubulo-glomerular feedback [31], leading to vasodilation of the afferent arterioles, with a consequent increase in renal blood flow, intraglomerular pressure and eGFR [28, 29]. However, while reductions in glomerular hyperfiltration may induce reductions in microalbuminuria and proteinuria levels, eGFR is expected to reduce [32]. There is limited knowledge concerning the longer-term effect of bariatric surgery on eGFR and CKD outcomes in patients with insulin-treated T2D. We have specifically investigated this patient cohort due to the negative effects of insulin on weight [4], a known predictor of adverse renal outcomes, as well as the fact that this group of patients are already at high risk of adverse-cardio-renal outcomes [5–8]. In addition, patients with diabetes are associated with accelerated loss of lean muscle mass [33], while bariatric surgery is associated with further loss of muscle mass and function [34]. Since markers of both muscle mass and strength being important predictors of outcomes in these patients with CKD [35], our study provided reassurance of the protective effects of bariatric surgery against progression of CKD.

The main strength of our study derives from the inclusion of a relatively large cohort of patients with T2D receiving insulin therapy who underwent bariatric surgery in a real-world population. In addition, our database is largely representative of the UK population, and as such, our findings will be generalizable to various populations that share similar demographics. The large cohort of patients studied here provides adequate statistical power and also contains information on other time-varying covariates to adjust for possible confounders. We adjusted for a large set of factors that could have differed at baseline. Nonetheless, some residual confounding in our study could persist. For example, our classification of albuminuria was largely based on a single measurement, in contrast to current recommendation, in which at least two measurements are required. Nonetheless, a single measure of urinary albumin within a large patient cohort provides a great deal of predictive information. In addition, as is the case in all studies of CV or ESRD risk associated with eGFR and albuminuria, the effect of competing hazards may bias estimates of risk. This is because elevated ACR and low eGFR are also risk factors for non-renal diseases, associated differential mortality in high-risk individuals may confound hazard ratio estimates for CV events. Lastly, changes after baseline in medications and subsequent changes in glycaemic indices or blood pressure were not evaluated in this analysis and, therefore, cannot account for any differences that might influence the association between ACR and outcomes.

Although bariatric surgery may protect patients who have diabetes with or without microalbuminuria against the risk of CKD, there is a modest protective effect on non-fatal CVD risk. Bariatric surgery also helps in overall improvement in renal outcomes such as eGFR. There is, however, a necessity for prospective investigation and appropriate investment for verifying or examining real-world effects of bariatric surgical intervention on renal function and stability of severely obese patients who are dependent on insulin treatment.

## Footnotes

1 Average value is calculated for multiple entry records that were found during the 90 days window for the same variable.
